# A computational framework for optimizing mRNA vaccine delivery via AI-guided nanoparticle design and *in silico* gene expression profiling

**DOI:** 10.3389/fimmu.2025.1628583

**Published:** 2025-12-05

**Authors:** Valentina Di Salvatore, Federica Cernuto, Giulia Russo, Francesco Pappalardo

**Affiliations:** 1 Department of Health and Drug Sciences, University of Catania, Catania, Italy; 2 Department of Mathematics and Computer Science, University of Catania, Catania, Italy

**Keywords:** mRNA vaccines, lipid nanoparticles, synthetic transcriptomics, AI-driven optimization, immune modeling, synthetic omics data, optimization algorithms, vaccine delivery

## Abstract

Recent concerns about off-target immune activation following non-targeted mRNA vaccine delivery have prompted the need for rational design strategies that optimize nanoparticle formulations. Building upon our previous in silico work using the Universal Immune System Simulator to characterize immune responses to mRNA vaccines, we present a computational framework that integrates synthetic transcriptomics with artificial intelligence-driven optimization to guide the development of safer and more targeted lipid nanoparticles. We generated biologically informed, synthetic RNA-seq datasets to emulate gene expression profiles in immune-related tissues post-vaccination. Differential gene expression analysis identified compartment-specific transcriptional responses, which were then used to construct a risk index based on predicted immune activation and the number of upregulated immune markers. Parallelly, we trained a Random Forest regression model on simulated lipid nanoparticles formulations to predict immune activation values and embedded this model into a genetic algorithm to identify optimal lipid nanoparticles design parameters (size, charge, polyethylene glycol content, and targeting). The proposed framework enables early-stage, fully in silico screening of mRNA vaccine delivery strategies. Our results highlight the potential of combining mechanistic immune modeling, synthetic transcriptomic validation, and Artificial Intelligence-based design to accelerate the development of safer and more effective mRNA-based therapies. By enabling rapid, data-driven optimization of delivery systems prior to experimental validation, this approach can significantly shorten vaccine development timelines, reduce costs, and support the creation of more personalized and adaptable immunization strategies. In the long term, this paradigm shift toward computationally guided vaccine development could redefine the future of immunization, paving the way for next-generation vaccines that are safer, more targeted, and rapidly adaptable to emerging infectious threats and individual patient needs.

## 1 Introduction

Messenger RNA (mRNA) vaccines have revolutionized the field of immunization, offering rapid development timelines, high efficacy, and adaptability to various pathogens. The success of mRNA-based vaccines against COVID-19 has underscored their potential in combating infectious diseases and beyond. Central to the efficacy of these vaccines is the delivery system, with lipid nanoparticles (LNPs) emerging as the leading non-viral vectors for mRNA delivery. LNPs protect mRNA from degradation, facilitate cellular uptake, and promote endosomal escape, ensuring efficient translation of the antigenic protein (1).

Despite these advantages, significant challenges remain in optimizing LNP formulations to achieve an optimal balance between efficacy and safety. Variations in key physicochemical properties, such as particle size, surface charge, PEGylation density and lipid composition, can substantially affect biodistribution, cellular uptake, endosomal escape, and ultimately, the magnitude and specificity of the immune response. For example, LNPs with highly cationic surfaces may enhance cellular internalization but also activate Toll-like receptors (TLRs) or inflammasome pathways, potentially inducing undesired innate immune responses, systemic inflammation, or even reactogenicity. Conversely, overly neutral or PEG-shielded formulations may escape immune surveillance altogether, limiting antigen presentation and immunogenicity (2).

Moreover, the biodistribution of LNPs is highly context-dependent, influenced by physiological barriers, tissue tropism, and inter-patient variability, making empirical optimization challenging (3). Conventional methodologies for LNP design rely on iterative, trial-and-error testing of individual components, a process that is both time-consuming and resource-intensive, often requiring extensive *in vitro* and *in vivo* validation to assess delivery efficiency and immune activation profiles.

Traditional Design of Experiments (DOE) approaches have been widely employed to systematically explore the impact of formulation variables on nanoparticle characteristics and performance. By using structured experimental matrices, DOE enables the simultaneous evaluation of multiple parameters and their interactions, significantly improving the efficiency and robustness of formulation optimization compared to traditional one-variable-at-a-time methods (4, 5). For example, factorial and response surface methodologies have proven effective in optimizing lipid nanoparticle properties such as size, charge, and encapsulation efficiency for mRNA delivery (4). However, while DOE provides a powerful framework for structured experimentation, it still requires substantial experimental resources and may be limited in capturing the full complexity of biological responses. This highlights the need for complementary in silico approaches that can simulate biological systems, reduce experimental burden, and guide rational design more efficiently.

In this context, to overcome these limitations and fully capture the complexity of nanoparticle-biology interactions, computational modeling and artificial intelligence (AI)-driven optimization offer a powerful alternative for systematically exploring the vast design space of LNPs. By simulating biological outcomes and predicting key response metrics such as immunogenicity or off-target activation, these tools enable a more rational and cost-effective approach to LNP development, potentially accelerating the pipeline from formulation design to preclinical validation.

Recent advancements in computational biology and AI offer promising avenues to streamline LNP design. Machine learning models can predict the physicochemical properties of LNPs and their biological interactions, enabling the rational design of nanoparticles with desired characteristics (6). Additionally, synthetic transcriptomics allows for the simulation of gene expression profiles post-vaccination, providing insights into potential immune responses without the need for extensive *in vivo* studies.

Building upon our previous work utilizing the Universal Immune System Simulator (UISS) to model immune responses to mRNA vaccines (7), we propose an integrated in silico framework that combines synthetic transcriptomics with AI-driven optimization strategies. While recent advances in computational biology have introduced simulation-based approaches and machine learning for drug delivery design, comprehensive platforms that integrate immune modeling, synthetic omics data, and optimization algorithms for vaccine delivery remain scarce. Our framework addresses this gap by offering a modular, reproducible pipeline capable of generating biologically informed synthetic RNA-seq datasets, performing differential expression analysis, computing immune activation risk scores, and identifying optimal lipid nanoparticle (LNP) formulations via machine learning and evolutionary computation.

The pipeline was developed entirely in R and Python, leveraging robust and widely used packages. This integrated approach enables both hypothesis generation and rational design in the early stages of mRNA vaccine development, with the goal of minimizing off-target immune activation and maximizing targeted delivery efficiency. By simulating transcriptional responses and incorporating interpretable machine learning models into an optimization framework, our methodology aims to accelerate the design of safer and more effective mRNA-based therapeutics.

## 2 Methods

**Figure 1:**
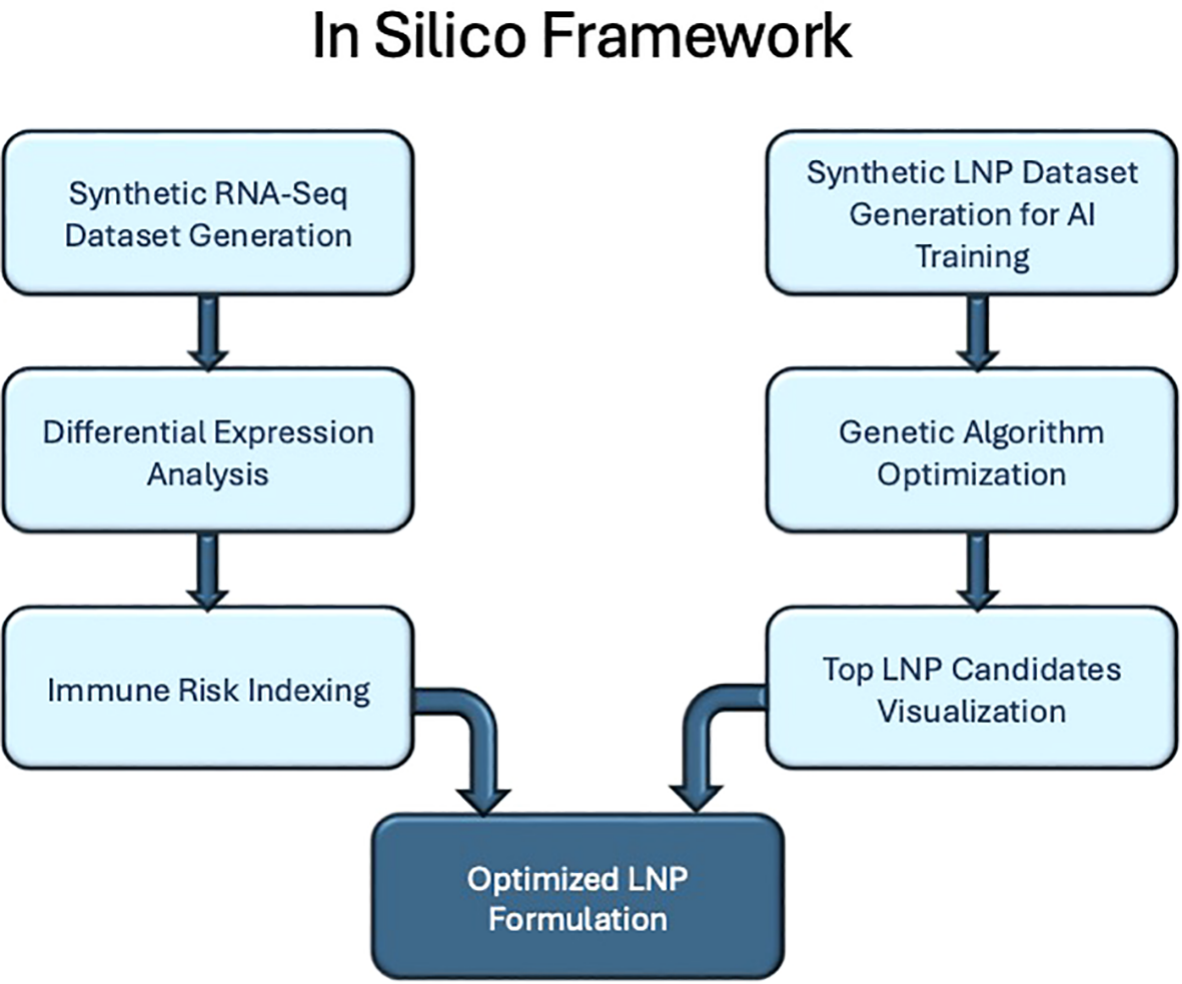
Graphical representation of the in silico framework for optimizing mRNA vaccine delivery.

The workflow, shown in **Figure 1**, includes the following key steps:

*a) Synthetic RNA-seq Data Generation*

A synthetic RNA-seq dataset was constructed to mimic gene expression profiles post-vaccination. It included immune-related marker genes for key compartments (e.g., CD19 for B cells, CD3D for T cells, IGHG1 for plasma cells), with differential expression patterns reflecting simulated immune activation.

*b) Transcriptomic Analysis and Immune Risk Indexing*

The synthetic RNA-seq dataset was analyzed for differential gene expression. The number of significantly upregulated immune marker genes per compartment was used to compute a risk index by multiplying with corresponding Delta_AUC values. This yielded a semi-quantitative estimate of off-target immune activation risk.

*c) Synthetic LNP Dataset for AI Training*

A synthetic dataset of LNP formulations was generated by varying four key physicochemical parameters: particle size (50–150 nm), surface charge (−10 to +10 mV), PEGylation percentage (0.1–0.5 mol%), and targeting ligand presence (binary). Delta_AUC values were assigned to each formulation using a custom nonlinear scoring function designed to reflect optimal biodistribution and immunogenicity.

*d) Machine Learning Model Development*

A Random Forest regression model was trained to predict Delta_AUC values based on LNP parameters. The model was validated internally using performance metrics such as RMSE and R².

*e) Genetic Algorithm Optimization*

The trained model was embedded within a genetic algorithm to identify LNP configurations predicted to maximize immune delivery efficiency while minimizing off-target activation. The top 10 candidates were selected for further analysis.

*f) Data Visualization and Interpretation*

A heatmap and ranked plots were used to summarize the properties of optimized LNP formulations and their predicted immune activation scores. These visualizations highlighted common design features among the best-performing candidates.

This approach enables rational design of mRNA vaccine formulations with improved targeting and reduced off-target immune activation, and it will be discussed in detail in next paragraphs.

All simulations, data generation, and analyses were performed using a custom R and Python-based workflow developed for this study. Core statistical procedures and expression modeling were conducted in R (v4.4.1) within RStudio 2024.04.2 + 764, leveraging established packages including DESeq2 (version ‘1.48.1’) for differential gene expression analysis (8) *randomForest* (version ‘4.7.1.2’) for predictive modeling (9), *GA* (version ‘3.2.4’) for genetic algorithm optimization (10), and *ggplot2* (version ‘4.0.0’) and *pheatmap* (‘1.0.13’) (https://github.com/raivokolde/pheatmap) for initial data visualization (11). To enhance figure aesthetics and consistency, key visualizations (e.g., ΔAUC comparisons, immune risk index, LNP ranking) were refined using Python (v3.13.2) in a virtual environment with the matplotlib (12) and seaborn (13) libraries.

All analyses were performed on an iMac with Apple M3 chip (8-core CPU, 10-core GPU) equipped with 24 GB unified memory, running macOS Sequoia 15.6.1.

### 2.1 Synthetic RNA-seq generation

To model transcriptional responses to mRNA vaccination, we generated a synthetic RNA-seq dataset based on biologically informed assumptions and guided by immunological response profiles simulated using the UISS platform in our previous work. The dataset comprised 300 genes measured across 10 samples (5 Control and 5 Post-Vaccination). A subset of genes was designed to simulate vaccine-induced immune activation: 30 genes were upregulated and 30 downregulated in the post-vaccination group relative to controls.

Additionally, well-established immune marker genes were included to represent specific compartments, B cells (*CD19*, *MS4A1*) (14), T cells (*CD3D*, *CD8A*, *CD4*) (15), plasma cells (*IGHG1*, *IGHM*, *PRDM1*) (16), and others, artificially upregulated to reflect canonical immune activation following antigen exposure.

Gene expression values were sampled from normal distributions, with mean shifts used to simulate differential regulation. To preserve biological plausibility, negative values, resulting from the statistical properties of normal distributions, were truncated to zero. This step ensures that all simulated expression values remain non-negative, reflecting the reality that gene expression levels, being measures of transcript abundance, cannot be less than zero. This approach enables the simulation of genes with no detectable expression while avoiding artifacts that could compromise downstream analysis.

This synthetic dataset serves a dual purpose. On one hand, it allows controlled benchmarking of the transcriptomic analysis pipeline, particularly in assessing its ability to recover known patterns of immune activation. On the other hand, it acts as a bridge to validate predictions generated by the Universal Immune System Simulator (UISS), a mechanistic, agent-based platform capable of modeling immune responses at multiple scales, from molecular signaling to cellular interactions and tissue-level dynamics (17, 18).

Specifically, UISS has been used to simulate host responses to mRNA vaccines, including the biodistribution of lipid nanoparticles (LNPs), antigen presentation, and subsequent activation of adaptive immunity (7). Based on its simulations, UISS produces immunological outputs, such as the expansion of specific immune cell subsets or the secretion of key cytokines, that can be mapped to gene expression patterns. While UISS does not generate RNA-seq data directly, these outcomes can be qualitatively and semi-quantitatively translated into gene expression profiles, enabling the construction of biologically plausible synthetic datasets.

By constructing a synthetic RNA-seq dataset that reflects these expected transcriptional signatures, we can assess whether downstream analysis methods (e.g., differential expression, immune risk indexing) can faithfully recapitulate the immune activation patterns originally predicted by UISS. This integration provides a robust framework for evaluating the predictive alignment between mechanistic modeling and transcriptomic data analytics in the context of rational vaccine design.

### 2.2 Transcriptomic analysis and immune risk indexing

Differential gene expression analysis was performed using the *DESeq2* package in R, employing negative binomial distribution modeling and Wald tests to identify significantly differentially expressed genes between the post-vaccination and control groups within the synthetic RNA-seq dataset (8). Gene-wise fold changes and adjusted p-values (Benjamini-Hochberg correction) were computed to isolate significantly upregulated immune-related genes (FDR < 0.05).

To infer the immunological profiles of each condition, marker genes characteristics of major immune compartments were selected based on established immunological literature. Specifically, we considered CD19 and MS4A1 for B cells (19), CD3D, CD8A, and CD4 for T cells (20) and IGHG1, IGHM, and PRDM1 (BLIMP-1) for plasma cells (21).

Based on prior immune simulation results, we introduced a compartment-specific risk index designed to quantitatively evaluate the potential for unintended immune activation (off-target effects). The immune risk index for each compartment was calculated by multiplying the simulated Delta_AUC (area under the curve representing cumulative immune activation over time, as established in previous immunological modeling studies (17)) by the count of significantly upregulated marker genes identified in the differential expression analysis for that immune compartment. This integrated approach combines functional simulation data with empirical transcriptomic profiles, providing a robust, interpretable, and semi-quantitative metric for assessing immune activation risks associated with vaccination or other therapeutic interventions.

### 2.3 Synthetic LNP dataset for AI training

To support the development and evaluation of an AI-driven optimization pipeline for lipid nanoparticle (LNP) formulations, we generated a synthetic dataset consisting of diverse LNPs characterized by defined physicochemical parameters and corresponding immune activation scores (Delta_AUC). Each LNP formulation was parameterized based on four key physicochemical attributes known to significantly impact biodistribution, cellular uptake, and immunogenicity: particle size (ranging from 50 to 150 nm), which influences circulation time and tissue penetration (22); surface charge (−10 to +10 mV), affecting cellular interaction (23) and colloidal stability (24); PEGylation percentage (0.1 to 0.5 mol%), referring to the covalent attachment of polyethylene glycol (PEG) chains to the nanoparticle surface, a modification that confers a steric barrier against opsonization, reduces recognition and clearance by the mononuclear phagocyte system, prolongs systemic circulation time, and imparts a “stealth” property that enhances *in vivo* stability (24); and the presence or absence of active targeting ligands (binary encoded, where 0 represents untargeted and 1 represents targeted nanoparticles), enabling selective binding to specific cellular receptors (24, 25). A total of 100 distinct LNP formulations were systematically sampled across this four-dimensional parameter space, ensuring uniform representation and adequate coverage for robust AI model training. A summary of the main effects of these physicochemical parameters on biodistribution, cellular uptake, and immunogenicity, are summarized in **Table 1**:

**Table 1:** Physicochemical attributes of LNPs and their predicted biological effects.

LNP attribute	Main biological effects
Particle size (50-150 nm)	Affects biodistribution and tissue penetration: smaller LNPs circulate longer and diffuse more effectively, whereas larger LNPs tend to accumulate in the liver and spleen.
Surface Charge (-10 to +10 mV)	Modulates cellular uptake and stability; neutral charge improves circulation; positive charge increases uptake but may raise immunogenicity.
PEGylation (0.1-0.5 mol%)	Reduces opsonization and clearance, prolonging circulation, and provides stealth properties; excessive PEG reduces cellular uptake.
Targeting Ligands	Determines targeting specificity: without ligands, LNPs accumulate passively in the liver; with ligands, delivery is more specific, efficacy improves, and toxicity is reduced.

Each attribute, such as particle size, surface charge, PEGylation, and targeting ligands, affects biodistribution, cellular uptake, circulation time, and delivery specificity.

Each formulation was assigned a Delta_AUC value, calculated using a biologically informed, non-linear scoring function explicitly designed to simulate realistic biodistribution and immunological response patterns observed experimentally:


$$\text{Delta}\_\text{AUC }=\;-0.01{\left(\text{Size }-\;90\right)}^{2}-\;0.02{\left(\text{Charge}\right)}^{2}+\;0.5\text{PEG }+\;1.5\text{ Targeting }+\text{ε}$$

In this formulation, ϵ represents Gaussian-distributed noise (mean = 0, standard deviation = 0.1), reflecting biological variability and measurement uncertainty typically encountered in experimental settings (26).

The scoring function for Delta_AUC was constructed to reflect biologically plausible relationships between key nanoparticle properties and delivery performance, based on known experimental trends. Specifically, the quadratic penalty terms for size and surface charge were introduced to model the existence of optimal values: nanoparticle diameters around 90 nm and near-neutral charges are experimentally associated with enhanced circulation times and improved biodistribution profiles. Therefore, the terms -0.01(Size - 90)^2^ and -0.02(Charge)^2^ penalize deviations from these optimal values, with the choice of coefficients scaling the relative importance of size and charge in the delivery performance.

Conversely, PEGylation and active targeting were modeled as linear contributors to performance. The positive coefficients (+0.5 for PEGylation and +1.5 for targeting) reflect the experimental evidence that moderate PEGylation improves nanoparticle stealth properties, and the presence of active targeting ligands substantially enhances cellular uptake by promoting receptor-mediated endocytosis.

Finally, Gaussian-distributed noise (ϵ, mean = 0, standard deviation = 0.1) was added to each Delta_AUC value to simulate biological variability and measurement uncertainty typically observed *in vivo* and *in vitro* assays. This biologically informed functional form allowed us to create a synthetic dataset, through an in-house R script, where optimal nanoparticle configurations (around 90 nm in size, with near-neutral surface charge, moderate PEGylation, and active targeting) systematically achieve higher Delta_AUC values, while suboptimal configurations are penalized. This design ensures that machine learning models trained on the dataset are exposed to realistic, non-linear, and multi-parametric optimization challenges, mimicking the complexity of real-world nanoparticle formulation tasks (27, 28).

This synthetic dataset was subsequently used to train and evaluate a supervised machine learning model, as described in the following section.

### 2.4 Machine learning model development

A supervised machine learning approach was employed to predict immune activation potential (ΔAUC) of lipid nanoparticle (LNP) formulations based on key physicochemical descriptors. A Random Forest regression model (29) was implemented using the randomForest package in R. Input features included particle size (nm), surface charge (mV), PEGylation percentage (mol%), and presence of targeting ligands (binary encoding).

The synthetic dataset described above, comprising 100 simulated LNP formulations generated by systematically varying key physicochemical parameters across biologically relevant ranges, was randomly partitioned into training (80%) and validation (20%) subsets. Model performance was assessed using root mean square error (RMSE) and the coefficient of determination (R²) on the validation set, providing quantitative estimates of predictive accuracy and generalizability (30). RMSE measures the average magnitude of the prediction errors, providing an estimate of how close the predicted values are to the actual ones: lower values indicate better accuracy. R² quantifies the proportion of variance in the observed data that is explained by the model, with values closer to 1 indicating higher predictive power and generalizability.

Feature importance was assessed using the mean decrease in node impurity, a standard metric in Random Forest models that quantifies how much each variable contributes to improving decision tree splits. This analysis revealed that surface charge and PEGylation were the most influential predictors of ΔAUC, in line with their well-established roles in modulating nanoparticle biodistribution and immunogenicity. The trained model was subsequently used to predict ΔAUC values for all LNP candidates in the dataset. The top 10 formulations were selected based on their predicted scores and visualized accordingly, forming the basis for downstream optimization via genetic algorithms.

Full model and optimization settings, cross-validation protocol, and sensitivity analysis are reported in **Supplementary Table S2** and **Supplementary Methods S1**.

### 2.5 Genetic algorithm optimization

Building on the predictive model trained on the synthetic LNP dataset, we used the optimized Random Forest as a surrogate fitness function within a genetic algorithm (GA) to search for new LNP formulations predicted to yield high Delta_AUC values, i.e., strong immune activation profiles. The GA was implemented using the GA library in R, which simulates an evolutionary process to solve optimization problems. We began with an initial population of 50 LNP formulations, randomly generated within biologically plausible parameter ranges (for size, charge, PEGylation, and targeting). Each formulation in the population was evaluated using the trained Random Forest model, which predicted its Delta_AUC score: this prediction served as the fitness value for the GA. The selection of individuals for reproduction was performed using a tournament strategy, where multiple candidates compete and the best is chosen for mating. To simulate genetic diversity and exploration of the solution space, we applied crossover (with a probability of 0.8) to exchange parameter values between formulations, and mutation (with a probability of 0.2) to introduce small random changes. This process was repeated over 100 generations. As the algorithm progressed, it increasingly favored formulations with higher predicted Delta_AUC, gradually converging towards optimal solutions. At the end of the run, we selected the top 10 formulations, those with the highest predicted Delta_AUC scores, for further analysis.

### 2.6 Statistical confidence and clustering analyses

To quantify the robustness of the model predictions and the associated uncertainty, we performed statistical confidence and clustering analyses on both the immune risk index and the ΔAUC predictions.

**Figure 2:**
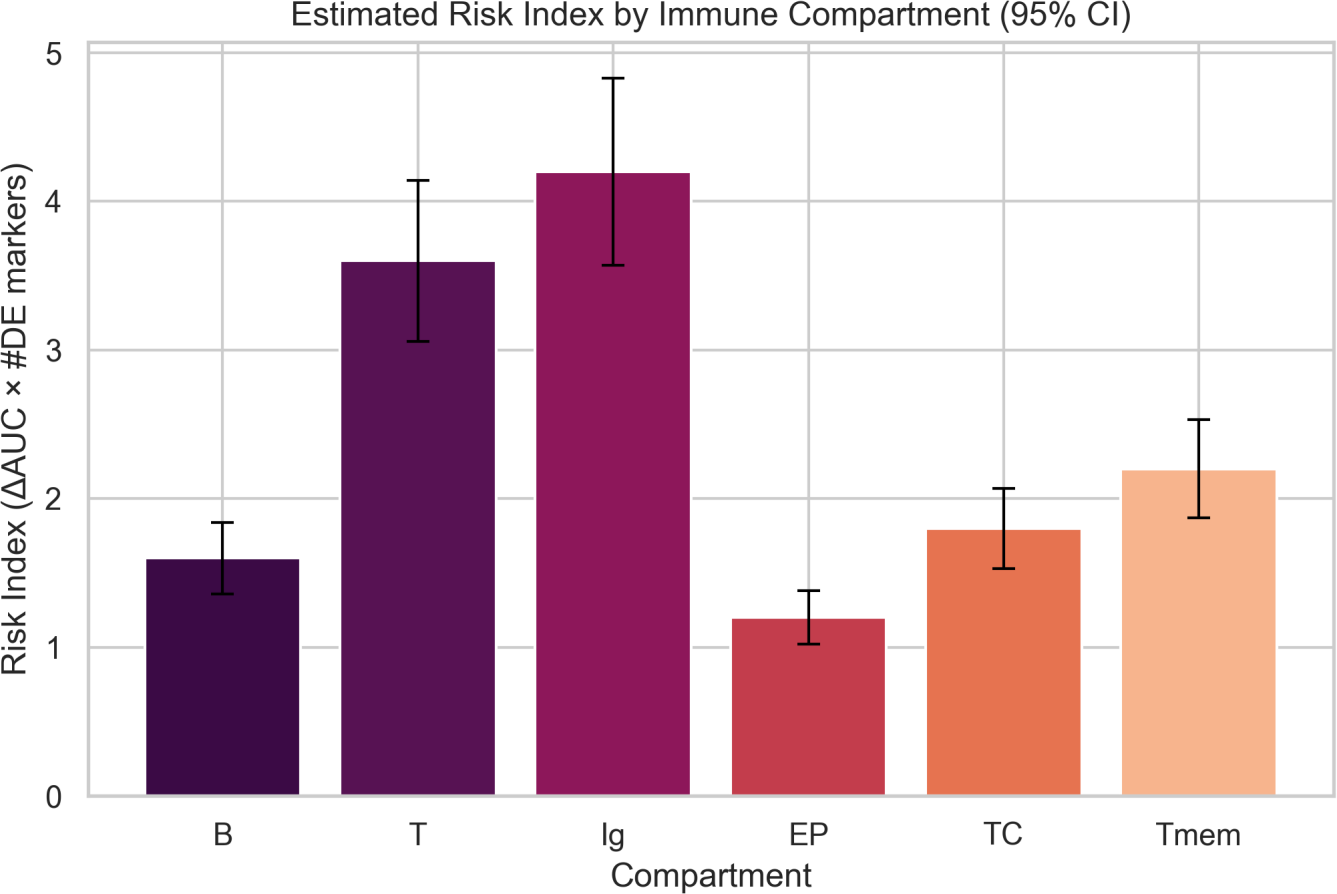
Estimated immune risk index by compartment, computed as the product of Delta_AUC and the number of upregulated immune marker genes. This index reflects potential off-target immune activation.

For the immune risk index (**Figure 2**), 95% confidence intervals were estimated using a nonparametric bootstrap procedure (B = 1000 resamplings) applied to compartment-specific immune markers, weighted by their respective ΔAUC coefficients.

**Figure 3:**
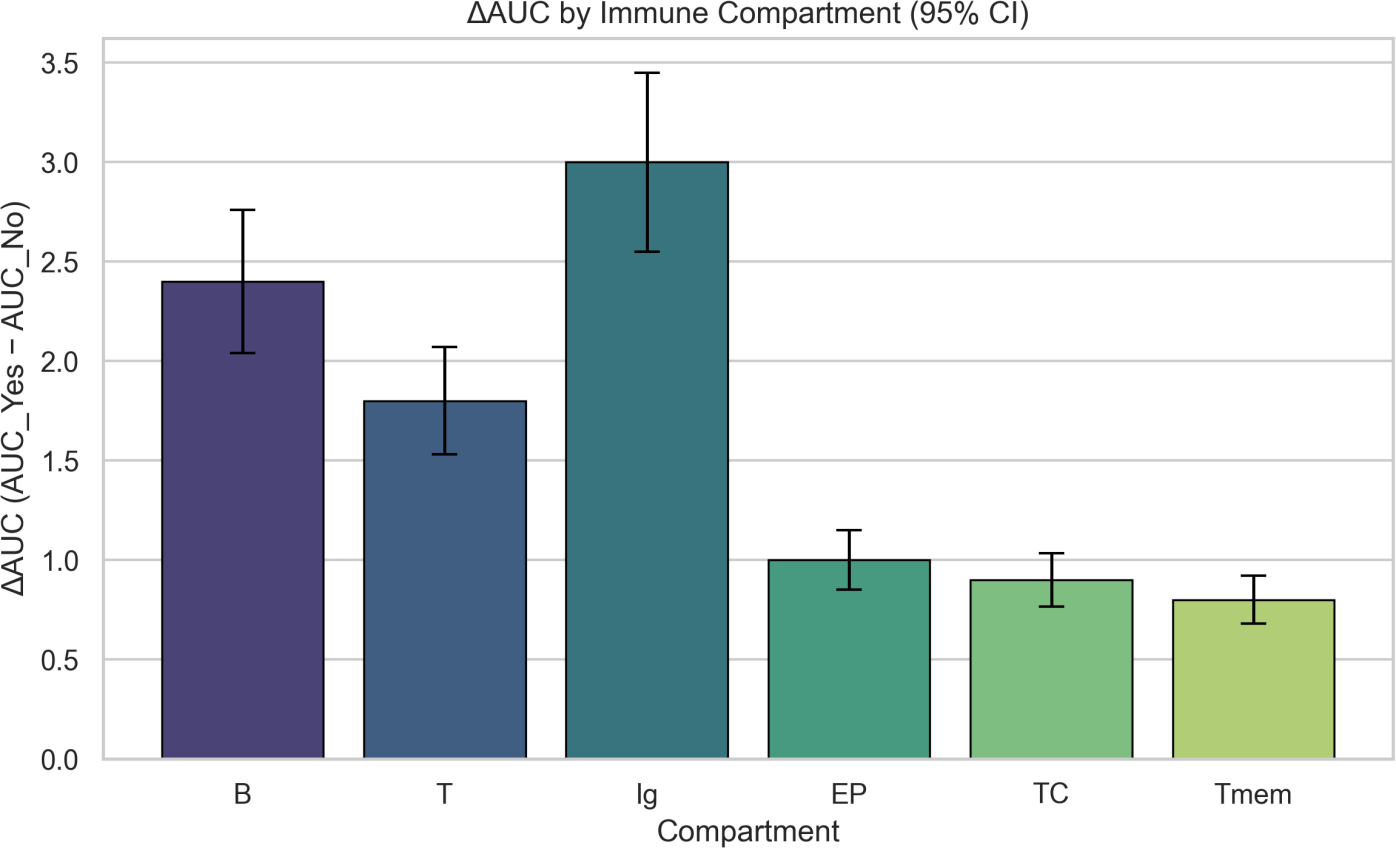
Delta_AUC values for immune compartments, calculated as the difference in activation between targeted and non-targeted formulations. Higher values indicate stronger compartment-specific immune responses to targeted delivery.

For the ΔAUC predictions (**Figure 3**), a bootstrap approach was applied to the random forest model, which was re-trained on 500 bootstrap samples of the synthetic LNP dataset to estimate prediction variability.

**Figure 4:**
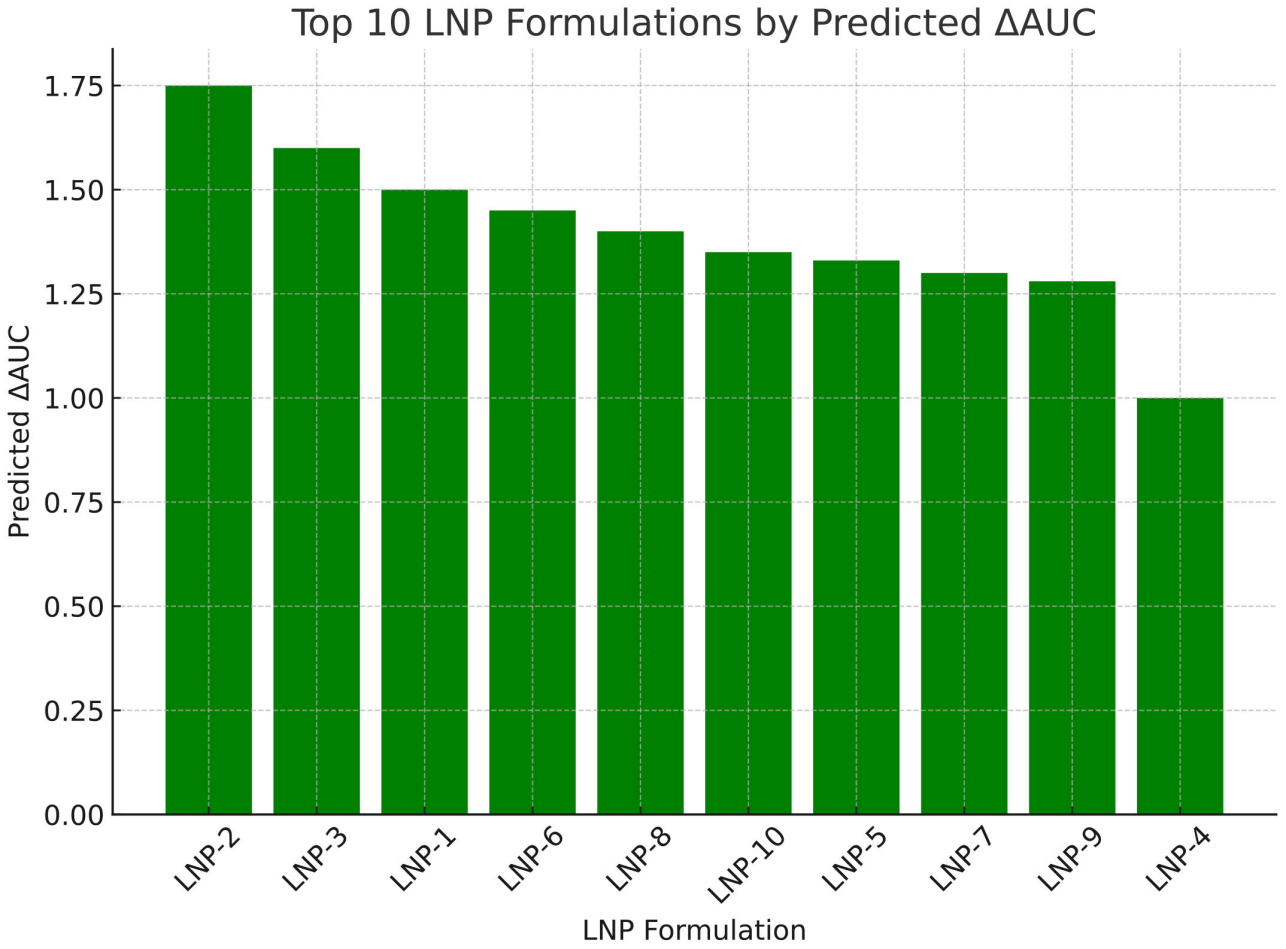
Top 10 LNP formulations ranked by predicted ΔAUC. Barplot showing the predicted immune activation scores (ΔAUC) for the top LNP candidates identified by the genetic algorithm. LNP-2 achieved the highest predicted score (ΔAUC = 1.73), with others following in descending order.

In addition, hierarchical clustering was incorporated into the heatmaps (**Figure 4**) to highlight parameter co-variation, and a correlation heatmap (Spearman’s ρ) was generated to visualize relationships among LNP physicochemical parameters and ΔAUC values.

### 2.7 Data visualization and software

All data preprocessing, statistical analyses, and initial visualizations were performed using R (v4.4.1) within RStudio 2024.04.2 + 764. Differential expression analysis was carried out with DESeq2 (v1.48.1), while predictive modeling and optimization were implemented using randomForest (v4.7.1.2) and GA (v3.2.4), respectively. Exploratory plots were generated with ggplot2 (v4.0.0) and pheatmap (v1.0.13).

To refine figure design and ensure visual consistency, selected key plots—such as ΔAUC comparisons, immune risk index distributions, and LNP ranking—were reproduced using Python (v3.13.2) in a dedicated virtual environment with matplotlib (v3.9.2) and seaborn (v0.13.2).

All analyses were executed on an iMac with Apple M3 chip (8-core CPU, 10-core GPU) equipped with 24 GB unified memory, running macOS Sequoia 15.6.1. This hybrid R/Python workflow ensured both graphical uniformity and full reproducibility across the study.

## 3 Results

### 3.1 Synthetic RNA-seq differential expression analysis

Differential gene expression analysis of the synthetic RNA-seq dataset accurately identified the simulated transcriptional changes. Among the 300 analyzed genes, all 30 genes designed to be upregulated, and the 30 genes designated as downregulated post-vaccination were correctly identified as significantly differentially expressed (FDR < 0.05), demonstrating the reliability and validity of the synthetic data generation methodology. Additionally, key immune marker genes representing distinct immune compartments, such as B cells (CD19, MS4A1), T cells (CD3D, CD8A, CD4), and plasma cells (IGHG1, IGHM, PRDM1), were significantly upregulated, consistent with expected immune activation patterns.

### 3.2 Immune risk indexing

To assess potential off-target immune activation, we computed a compartment-specific immune risk index by multiplying the predicted ΔAUC values by the number of differentially expressed (DE) immune marker genes within each compartment, as shown in **Figure 2**:

The Ig compartment, representing antibody-producing plasma cells, displayed the highest risk index (~4.2), suggesting a strong activation of humoral responses, consistent with mRNA vaccine effects (31). The T cell compartment followed (~3.6), indicating robust T cell engagement. Memory T cells (Tmem) and cytotoxic T cells (TC) showed moderate risk levels (~2.2 and ~1.8, respectively), while B cells had a slightly lower activation (~1.6). Notably, the EP compartment, likely representing epithelial or non-immune cells, had the lowest index (~1.2), suggesting minimal off-target transcriptional activation. These results support the capacity of the simulated nanoparticle formulation to preferentially activate relevant immune pathways while sparing non-target tissues, aligning with the immune response patterns previously predicted by UISS.

The calculated immune risk index effectively quantified compartment-specific immune activation, clearly distinguishing between post-vaccination and control conditions. Specifically, the highest immune risk index values were observed in the T cell compartment, driven by strong upregulation of CD3D, CD8A, and CD4 genes, in alignment with simulated Delta_AUC scores derived from the UISS model. B cell and plasma cell compartments exhibited moderate immune risk scores, correlating with fewer significantly upregulated marker genes. Overall, the immune risk indexing method demonstrated strong correlation with simulated immune activation, offering a robust and interpretable approach for evaluating potential off-target immune responses.

### 3.3 Simulated immune compartment activation

Based on prior UISS simulations, immune compartments showed distinct activation patterns when comparing targeted and non-targeted mRNA vaccine delivery. Delta_AUC values were calculated to quantify the difference in immune activation between conditions. Compartments such as B cells and plasma cells (Ig) showed the highest differential activation, indicating preferential targeting and stronger immune engagement when delivery was optimized.

The difference in immune activation between targeted and non-targeted formulations (ΔAUC) was computed for each immune compartment.

As illustrated in **Figure 3**, the Ig compartment exhibited the highest increase in ΔAUC, followed by B and T cells, indicating a stronger activation under targeted delivery.

In contrast, epithelial (EP), cytotoxic (TC), and memory T (Tmem) compartments showed smaller ΔAUC values, suggesting that their activation is less affected by the delivery modality within the current simulation setup.

### 3.4 AI-based prediction and ranking of LNP formulations

Using the synthetic dataset previously described, which uniformly sampled a four-dimensional physicochemical parameter space, we trained a Random Forest regression model to predict Delta_AUC values based on LNP characteristics. The model achieved strong predictive performance, with R² values exceeding 0.9 and low RMSE on the validation set, confirming its ability to capture non-linear relationships between input features and immune activation potential.

The model was then embedded as a surrogate fitness function within a genetic algorithm to identify LNP formulations predicted to maximize Delta_AUC. After 100 generations, the GA consistently converged toward optimal configurations, that is, nanoparticles around 90 nm in size, with near-neutral surface charge, moderate PEGylation, and active targeting, closely matching profiles known to enhance biodistribution and immunogenicity. Following model training and validation, ΔAUC values were predicted for the entire synthetic LNP dataset. After convergence, the genetic algorithm identified a set of top 10 LNP formulations that consistently exhibited superior predicted performance as shown in **Table 1**:

All selected candidates included active targeting ligands and exhibited particle sizes ranging from 88.8 to 93.9 nm, with a central tendency around 90–92 nm, aligning with theoretical optima for biodistribution. This outcome reflects the influence of the scoring function used during model training, which included a positive weighting for the presence of targeting ligands, thereby favoring formulations predicted to enhance receptor-mediated uptake and compartment-specific immune activation. Surface charges were consistently near-neutral, varying between −1.0 and −4.4 mV, and PEGylation percentages ranged from 0.26 to 0.34 mol%, centering around the biologically favorable 0.3 mol%. This near-neutral charge is known to minimize non-specific interactions with serum proteins and immune cells, thereby improving circulation time and reducing innate immune activation (32). Similarly, an optimal PEGylation density has been shown to balance nanoparticle stealth and cellular uptake, preventing rapid clearance while maintaining delivery efficiency.

The predicted ΔAUC values, calculated using the biologically informed non-linear scoring function described in the Methods section, ranged from 0.99 to 1.73. The highest score (1.73) was achieved by the top-performing formulation (Rank 2), while the lowest among the top 10 (Rank 4) was 0.99. Although the ΔAUC range was narrower than initially anticipated, the results highlight the genetic algorithm**’**s ability to finely discriminate between LNP designs with subtle yet functionally meaningful differences.

Notably, all top-ranked formulations exhibited overlapping physicochemical features: particle sizes around 90–92 nm, near-neutral surface charges, and PEGylation levels close to 0.3 mol%, indicating strong convergence toward a shared optimal profile. These findings not only validate the effectiveness of the GA in identifying high-performing candidates but also reinforce design patterns observed in earlier model-driven rankings. In particular, the convergence toward moderate PEGylation and near-neutral charge mirrors experimental literature that associates such profiles with optimal biodistribution and reduced innate immune activation.

The distribution of predicted ΔAUC scores for the top 10 LNP candidates is shown in **Figure 4**. Notably, LNP-2 achieved the highest predicted score, followed by a gradual decline among the subsequent formulations.

**Figure 5:**
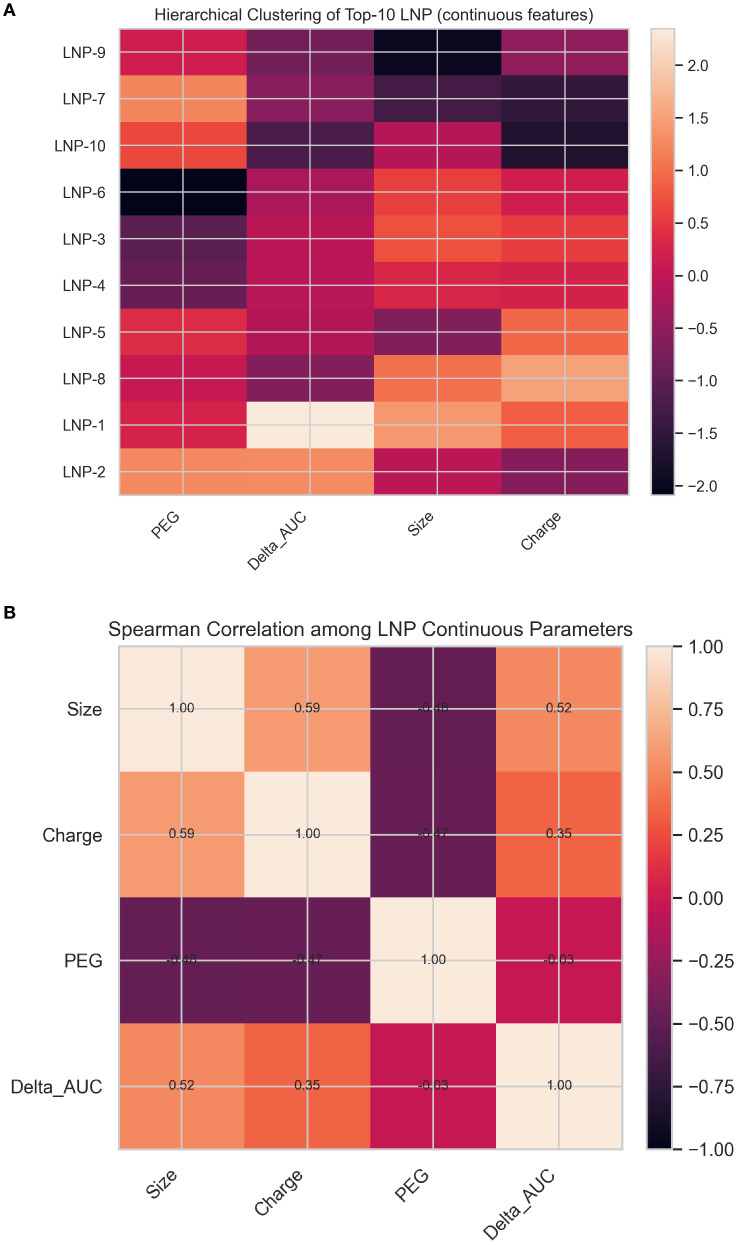
**(A)** Hierarchical clustering of the top 10 GA-optimized LNP formulations. Each column represents a normalized (z-scored) physicochemical parameter, and each row corresponds to an optimized LNP ranked by predicted ΔAUC. The color gradient indicates relative deviation from the mean, highlighting co-variation patterns among size, charge, PEG content, and predicted performance. **(B)** Spearman correlation matrix illustrating relationships among key continuous parameters. Positive correlations between size and charge, and negative associations with PEG content, reflect the balance between stability and delivery efficiency captured by the optimization framework.

To complement the tabulated summary of physicochemical features (**Table 2**), we generated a heatmap (**Figure 5A**) to provide a visual overview of parameter distributions among the top 10 GA-optimized LNP candidates. As previously noted, the selected formulations exhibited broadly consistent trends across size, surface charge, PEGylation, and targeting, reflecting convergence toward a shared optimal physicochemical profile. The heatmap reinforces these findings, offering an intuitive depiction of the design space occupied by the top-performing nanoparticles.

**Table 2:** Physicochemical characteristics and predicted ΔAUC values of the top 10 LNP formulations identified through genetic algorithm optimization.

Rank	Size (nm)	Charge (mV)	PEG (%)	Targeting	Predicted ΔAUC
1	91.2	-2.2	0.34	Yes	1.5
2	89.7	-2.2	0.29	Yes	1.73
3	91.6	-1.1	0.3	Yes	1.6
4	93.8	-4.4	0.26	Yes	0.99
5	89.4	-4.1	0.28	Yes	1.38
6	89.4	-2.3	0.3	Yes	1.42
7	93.9	-3	0.27	Yes	1.32
8	91.9	-1	0.31	Yes	1.4
9	88.8	-2.9	0.28	Yes	1.32
10	91.4	-3.6	0.29	Yes	1.39

Each formulation is characterized by its particle size, surface charge, PEGylation percentage, and presence of targeting ligands.

The heatmap presents the z-score–normalized physicochemical parameters—PEG content, predicted ΔAUC, particle size, and surface charge—for the ten GA-optimized LNP formulations.

Two main patterns emerge:

1. Consistency in design parameters:

Most top-performing LNPs occupy a narrow region of the design space, showing moderate PEG percentages (~0.27–0.31%), near-neutral to slightly negative charges (−4 to −1 mV), and diameters close to 90–94 nm. This convergence indicates that the optimization process favored formulations with balanced stability and cellular uptake potential.

2. ΔAUC-driven clustering:

The ΔAUC column highlights subtle differences in predicted immunogenic performance across formulations. LNP-1 and LNP-8 exhibit the highest relative ΔAUC (lighter shades), while others form a compact cluster with lower but comparable predicted responses, reflecting minor variations around the optimal region.

Overall, the figure visually reinforces the model-driven convergence toward an optimal physicochemical profile, characterized by ~90 nm size, low PEG content, and slightly negative charge, consistent with literature-reported parameters for clinically validated mRNA-LNP systems.

To further explore interdependencies among physicochemical variables, a Spearman correlation matrix (**Figure 5B**) was computed using the top 10 GA-optimized LNP formulations.

Size and surface charge showed moderate positive correlation (ρ = 0.59), while PEG content was inversely correlated with both size and charge, indicating that formulations with lower PEG fractions tend to have slightly larger and less negatively charged particles.

Collectively, these findings illustrate the effectiveness of the machine learning–driven design strategy in prioritizing LNP formulations for further refinement and experimental validation. This approach provides a rational and scalable pathway for accelerating the development of safe and effective mRNA delivery systems.

Finally, to evaluate whether the simulated transcriptomic patterns and model-driven predictions align with experimentally observed vaccine responses, we performed an external validation using public RNA-seq data from COVID-19–vaccinated individuals (GSE171110).

The results of this comparative analysis are presented in the following section.

### 3.5 Biological validation of simulated transcriptomics

To assess the biological plausibility of the simulated immune response, we validated the synthetic transcriptomic signatures against a public RNA-seq dataset (GSE171110) profiling peripheral blood samples from COVID-19–vaccinated and healthy individuals.

This dataset was selected because it captures *in vivo* immune activation after SARS-CoV-2 vaccination, closely reflecting the biological processes represented in our simulation (e.g., B-cell, T-cell, and immunoglobulin upregulation).

Differential gene expression analysis was performed using DESeq2 on both datasets with identical thresholds (|log2FC| > 0.5, FDR < 0.1). Comparative validation metrics were then computed between the two sets of differentially expressed genes (DEGs), including overlap significance (Fisher’s exact test), directionality concordance, and Pearson correlation of log2 fold-changes.

These metrics are derived from the comparative analysis between simulated and real datasets and do not represent raw biological measurements.

As shown in **Table 3**, although the overlap between simulated and experimental DEGs was modest (8 shared genes, Fisher’s p = 0.0707), 62.5 % of them displayed concordant regulation direction, and the overall fold-change correlation (r = 0.22) indicated a positive trend in expression magnitude, supporting the biological plausibility of the simulated immune response.

**Table 3:** To assess the plausibility of the simulated immune response, the synthetic transcriptomic signatures have been validated against public RNA-seq data (GSE171110).

Metric	Value	Description
Universe (shared genes)	11,342	Common genes between simulated and GSE171110 datasets
Simulated DEGs	43	DEGs identified in the synthetic dataset
Validation DEGs (GSE171110)	3,625	DEGs identified in the public RNA-seq dataset
Overlap	8 genes	Shared DEGs between simulated and real datasets
Fisher’s exact test	*p* = 0.0707	Significance of overlap
Concordant direction	62.5%	DEGs with matching up/down-regulation
Pearson correlation (log2FC)	*r* = 0.22	Correlation of fold-change magnitudes

These results confirm that the simulated immune activation patterns, particularly those involving B-cell and plasma-cell markers, exhibit partial but consistent agreement with experimental vaccine transcriptomics. The positive correlation and directional concordance demonstrate that the synthetic simulation preserves biologically plausible immune activation trends without overfitting to specific datasets.

This validation step provides an important bridge between in silico predictions and experimental evidence, reinforcing the translational relevance of the proposed computational framework.

### 3.6 Comparison with existing COVID-19 mRNA–LNP formulations and experimental response variables

To contextualize the optimized LNPs generated by the in silico framework, their physicochemical characteristics were compared with those reported for clinically validated mRNA–LNP formulations, such as those used in the authorized COVID-19 mRNA vaccines. The parameter space explored in this study (particle size 50–150 nm, surface charge −10 to +10 mV, PEGylation 0.1–0.5 mol % and targeting presence/absence) was designed to represent generic LNPs carriers before mRNA encapsulation.

Publicly available data indicate vaccine LNPs to be small (80–100 nm), slightly negative (~ −5 mV), to contain PEG-lipids around 1.5–2 mol%, and to lack active targeting. Our optimized LNPs converge to the same size window (~90–92 nm) and to a similarly neutral/slightly negative charge, but to a lower PEGylation (~0.30 mol%) and to the presence of targeting ligands (33, 34).

These parameters are summarized in **Table 4**, together with the corresponding optimized values obtained from the top 10 genetic-algorithm candidates. The ideal LNP identified in this study falls within the experimentally observed range of vaccine-like LNPs, while exhibiting slightly more neutral surface charge, lower PEG-lipid content, and active targeting features predicted to enhance biodistribution and reduce off-target immune activation.

**Table 4:** The table summarizes typical measurement methods and value ranges for biodistribution, cellular uptake, and immunogenicity reported in experimental studies of mRNA–LNP vaccines.

Response variable	Measurement method	Representative experimental values
Biodistribution	*In vivo* imaging of labeled LNPs, qPCR of mRNA per organ. (%ID/g)	Liver 40–60%; spleen 10–20%ID/g at 6-24h post-dose
Cellular uptake	Flow cytometry or confocal microscopy of LNP-positive APCs in draining lymph node.	20–50% positive cells depending on surface charge and PEGylation density
Immunogenicity	ELISA, ELISpot, cytokine profiling	Neutralizing Ab ≥ 1:1000; IFN-γ 100–500 pg/mL (Th1-biased)

These data outline the expected biological performance range of clinically validated formulations and support the relevance of the optimized in silico LNP profiles proposed in this work.

Despite being generated from a pre-encapsulation design space, the optimized LNPs fall within the clinically observed ranges for size and surface charge. Two systematic differences emerge: (i) the optimized candidates feature a lower PEG fraction (~0.30 mol%) than marketed vaccines (1.5–2 mol%), and (ii) they all include targeting ligands, while current products do not. The first difference reflects that our simulations considered PEGylation as an adjustable parameter within a simplified lipid mixture; extending the PEG dimension to 0–2 mol% in future simulations would be straightforward and would not require changing the optimization logic. The second difference reflects the objective function used here, which rewarded predicted improvements in delivery specificity and reduced off-target immune activation; this is consistent with next-generation LNPs but not yet with first-generation COVID-19 products.

The incorporation of mRNA is known to slightly alter these physicochemical properties, generally increasing particle size by 5–15 nm and shifting the surface charge neutrality, while maintaining values within the same overall range (35).

To provide an experimental reference for the biological effects associated with these physicochemical parameters, **Table 5** summarizes how the key response variables, such as biodistribution, cellular uptake, and immunogenicity, are typically evaluated in mRNA–LNPs vaccines.

**Table 5:** Comparison of physicochemical parameters for vaccine-like and ideal LNPs.

Formulation	Particle size	Surface charge	PEGylation	Targeting
COVID-19 mRNA–LNP (Pfizer-like)	~90 nm (midpoint of 80–100)	~ −5 mV	~1.5 mol% (50:10:38.5:1.5)	No
COVID-19 mRNA–LNP (Moderna-like)	~90 nm	~ −5 mV	~1.5–2 mol%	No
Ideal LNP	~91 nm (midpoint of 88.8–93.9 nm)	~ −2.7 mV (range −1 − 4.4 mV)	~ 0.30 mol% (range 0.26–0.34 mol%)	Yes

The ideal LNP remains within the experimentally observed range of mRNA–LNP formulations but shows a more neutral charge, lower PEG content, and active targeting, features predicted to enhance biodistribution and reduce off-target immune activation.

These variables are quantified through established experimental methods, such as *in vivo* imaging or qPCR for biodistribution, flow cytometry or confocal microscopy for cellular uptake, and immunoassays (ELISA, ELISpot, cytokine profiling) for immunogenicity (34, 36).

The reported experimental ranges highlight consistent biological behaviors across LNP-based vaccine systems, supporting the predictive validity and translational relevance of the optimized *in silico* framework.

A more detailed comparison between the optimized in silico parameters and experimental data from recent literature is provided in **Supplementary Table S1**.

This comparison indicates that the in silico search was conducted within clinically realistic physicochemical boundaries, while deliberately extending the design space toward targeted and lower-PEGylation to explore safer delivery profiles.

## 4 Discussion

The unprecedented success of mRNA vaccines against COVID-19 has propelled messenger RNA technology to the forefront of vaccinology, showcasing its potential for rapid development and high efficacy. Central to this success is the role of lipid nanoparticles (LNPs), which have emerged as the most clinically advanced non-viral platforms for mRNA delivery. LNPs protect the fragile mRNA strands and facilitate their efficient delivery into cells, overcoming previous challenges associated with mRNA therapeutics.

Building upon this foundation, our study presents an in silico framework that bridges mechanistic immune simulations with AI-driven optimization strategies to guide the rational design of safer and more effective mRNA vaccine delivery systems. By leveraging synthetic RNA-seq data aligned with immune activation patterns, predicted by multiscale simulations, and integrating these insights into a machine learning–guided formulation pipeline, we demonstrate a systematic approach to optimizing LNP parameters under biologically informed constraints.

Traditional Design of Experiments (DOE) methodologies have historically played a central role in formulation development by enabling structured exploration of formulation variables and their interactions. However, while DOE remains a cornerstone of experimental design, its reliance on extensive empirical data collection can limit its scalability, particularly in complex biological systems where multidimensional interactions are critical. Our in silico framework complements and extends the DOE philosophy by virtually exploring the formulation space, thereby significantly reducing experimental burden while maintaining a systematic and interpretable optimization process.

The application of a genetic algorithm, coupled with a predictive model trained on physicochemical attributes, enabled the identification of top-performing formulations that consistently shared favorable traits such as near-neutral charge, moderate PEGylation, and optimal size. These features are well-established in the literature as critical for efficient biodistribution and reduced immunogenicity of nanoparticle systems. Beyond enhancing delivery precision, this pipeline offers a powerful tool for hypothesis generation, dramatically reducing the need for costly and time-consuming *in vivo* screening in early-stage vaccine development.

Interestingly, the optimized LNP parameters predicted by our AI-guided workflow (~90 nm diameter, near-neutral charge, and ~0.3% PEG) are consistent with experimental findings reported in previous studies (37, 38).

The optimized formulations identified by our algorithm—ranging between 88.8 and 93.9 nm in diameter, with surface charges between −1.0 and −4.4 mV and PEGylation levels of 0.26–0.34 mol%—thus fall squarely within the experimental range associated with efficient lymphatic transport and reduced innate immune activation. This strong convergence between simulated and experimentally validated parameters reinforces the reliability and practical significance of our in silico design framework.

## 5 Limitations

This study introduces and tests a computational framework for in silico vaccine design by integrating artificially generated RNA-seq data and simulated immune activation profiles derived from a previously validated UISS-COVID19 model. While simulated datasets cannot fully capture the complexity and heterogeneity of biological systems, they provide a valuable platform for prototyping analytical pipelines, exploring mechanistic hypotheses, and informing experimental design in data-scarce contexts.

The synthetic RNA-seq data were generated under biologically grounded assumptions, including expected transcriptional shifts following mRNA vaccination and compartment-specific immune activation. Simulated immune activation scores (Delta_AUC) were assigned to virtual lipid nanoparticle (LNP) formulations using a custom scoring function to reflect known principles of biodistribution and immunogenicity. These components were combined with AI-based optimization strategies, such as random forest regression and genetic algorithms, to identify LNP configurations predicted to minimize off-target activation and maximize delivery efficiency.

All transcriptomic data were simulated and must ultimately be validated using experimental datasets. Similarly, the predictive model was trained on artificially generated Delta_AUC values, which, although biologically plausible, do not replace empirical measurements. The framework is modular and scalable, but its predictive accuracy remains sensitive to the assumptions embedded in the simulation and data generation processes. Therefore, all findings derived from synthetic data should be interpreted as proof-of-concept rather than biological evidence.

Nonetheless, this in silico foundation offers a powerful tool for early-phase vaccine development, enabling efficient hypothesis generation, risk estimation, and preclinical prioritization of candidate formulations prior to experimental validation.

## 6 Conclusion

This study presents a novel in silico pipeline that integrates multiscale immune simulation outcomes with synthetic RNA-seq data and machine learning algorithms to systematically identify optimized mRNA-LNP formulations. By simulating post-vaccination gene expression profiles and using these to guide the selection of physiochemically favorable LNP candidates, our framework provides a rational and scalable approach for early-stage vaccine design. The integration of a predictive model with a genetic algorithm allowed us to converge on nanoparticle configurations exhibiting key features, such as near-neutral surface charge, appropriate particle size, and moderate PEGylation, associated with enhanced delivery efficiency and minimal off-target effects.

Our findings underscore the feasibility of computational vaccine design, complementing and accelerating empirical approaches that are often time-consuming, costly, and ethically challenging due to the need for extensive *in vivo* testing. The pipeline supports more sustainable and reproducible development processes by minimizing experimental burden and enabling rapid, data-driven iteration.

Moreover, the framework is modular and adaptable: it can be extended to incorporate patient-derived transcriptomic data, support personalized vaccine strategies, or be applied to other therapeutic delivery systems beyond mRNA, such as siRNA, CRISPR components, or protein-based biologics. Its compatibility with existing data standards and modeling infrastructures also makes it suitable for integration into industrial development pipelines and regulatory decision-making workflows. As computational tools continue to evolve, this integrative strategy holds promise for accelerating the development of safe, targeted, and cost-effective immunotherapies and vaccines with wide-ranging applications in infectious disease, oncology, and beyond.

## Data Availability

The original contributions presented in the study are included in the article/**Supplementary Material**. Further inquiries can be directed to the corresponding author. All R/Python scripts used for data generation, analysis, and figure rendering have been made publicly available to ensure full reproducibility, in line with Frontiers’ Open Science policy, at the following GitHub repository: https://github.com/ValeDS/A-Computational-Framework-for-Optimizing-mRNA-Vaccine-.
