# Supplementary figures and images for: A computational framework for optimizing mRNA vaccine delivery via AI-guided nanoparticle design and in silico gene expression profiling
Source: Front Immunol. 2025 Dec 5;16:1628583. doi: 10.3389/fimmu.2025.1628583 (PMC12714931; doi:10.3389/fimmu.2025.1628583)

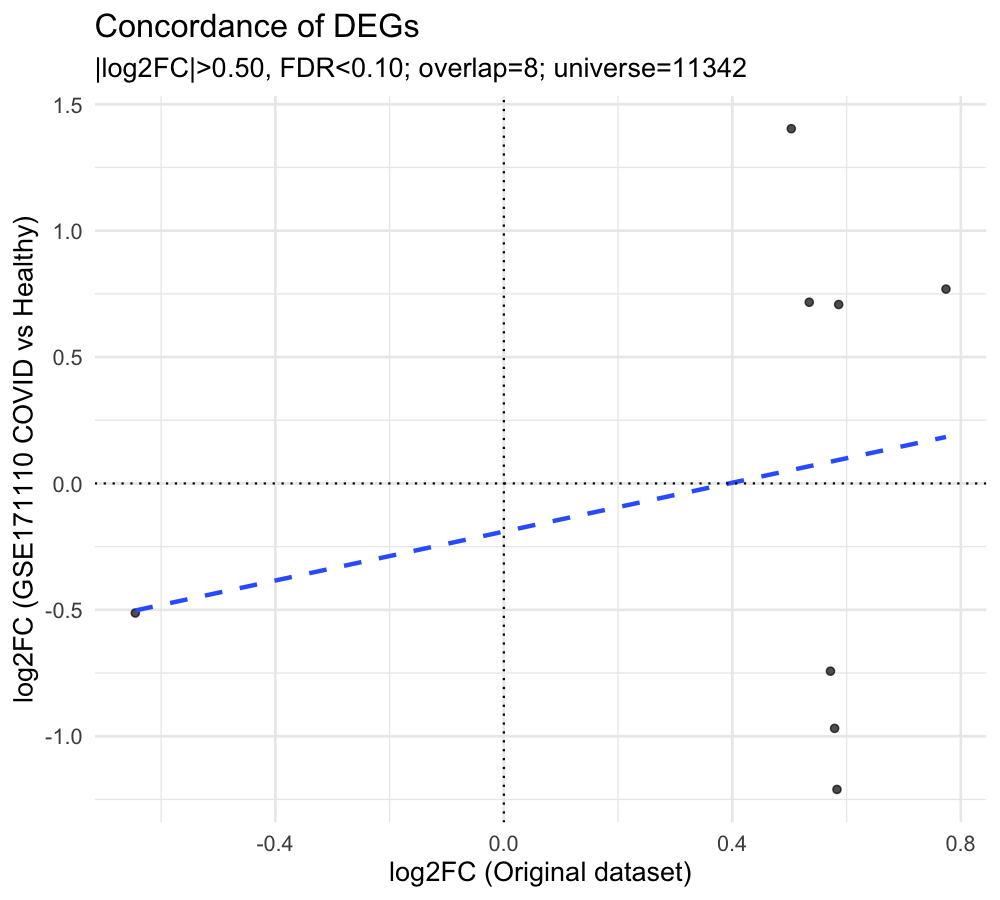

Supplement: Supplementary file 1 [file Image1.png]

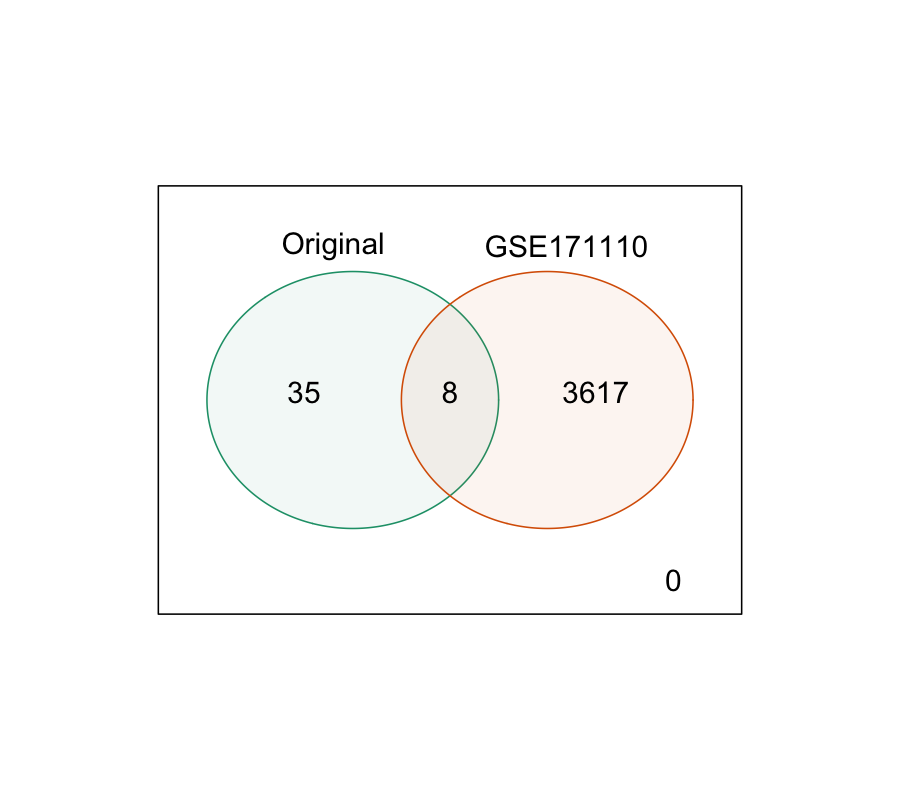

Supplement: Supplementary file 2 [file Image2.png]
