# Supplementary material for: A computational framework for optimizing mRNA vaccine delivery via AI-guided nanoparticle design and in silico gene expression profiling
Source: Front Immunol. 2025 Dec 5;16:1628583. doi: 10.3389/fimmu.2025.1628583 (PMC12714931; doi:10.3389/fimmu.2025.1628583)
Supplement: Supplementary file 3 [file Presentation1.pdf]

Supplementary Methods S1 — Cross-validation, Genetic Algorithm, and Sensitivity Analysis

Cross-validation.

The Random Forest (RF) model was trained on 80% of the synthetic dataset and evaluated using **5-fold cross-validation** on the training set, followed by performance assessment on a **20% hold-out validation subset**.

The cross-validation yielded **$R^2 \approx 0.87$** with consistent RMSE values, while the independent validation set achieved **$R^2 > 0.9$**, confirming model robustness and generalizability (see accompanying notebook on GitHub).

Genetic Algorithm.

The GA optimization was implemented using the GA::ga package in R, initialized with a **Latin-hypercube–sampled population** spanning the four key parameters (particle size, surface charge, PEGylation, and targeting). Default elitism was applied, with the **fitness function defined as the RF-predicted ΔAUC**.

The algorithm ran for **100 generations** with a population size of 50, producing convergence toward stable optima. The **top 10 formulations** identified are reported in the main manuscript (Table 2).

Sensitivity Analysis.

A **Sobol' variance decomposition (Saltelli scheme)** was applied to the four-parameter design space to quantify each factor's contribution to ΔAUC variance. The relative contributions (Size = 0.41, Charge = 0.32, PEG = 0.17, Targeting = 0.10) are summarized in **Supplementary Table S2**. The analysis was performed in R using standard implementations.
