# Supplementary material for: A computational framework for optimizing mRNA vaccine delivery via AI-guided nanoparticle design and in silico gene expression profiling
Source: Front Immunol. 2025 Dec 5;16:1628583. doi: 10.3389/fimmu.2025.1628583 (PMC12714931; doi:10.3389/fimmu.2025.1628583)
Supplement: Supplementary file 4 [file Table1.pdf]

Table S1. Comparison between in silico optimized LNP parameters and experimental formulations reported in recent literature.

Parameter	Experimental values	In silico optimized results (this study)	Interpretation	Supporting reference(s)
Particle size (nm)	*Hassett et al.:* effective vaccine formulations centered ≈ 90 nm (analyzed over 800–10,000 nm range using 30 µm aperture). *Kong et al.:* 94.6 ± 1.1 (LNP-S), 121.9 ± 1.6 (LNP-M), 167.4 ± 1.9 (LNP-L).	88.8 – 93.9 nm (mean ≈ 91 nm)	Excellent convergence—both experimental and simulated data identify ≈ 90 nm as optimal for lymphatic drainage, biodistribution, and antigen expression.	Hassett et al., *Nat Nanotechnol*, 2021; Kong et al., *J. Nanobiotechnol*, 2024
Polydispersity index (PDI)	*Kong et al.:* 0.13 ± 0.02 (LNP-S) → monodisperse. *Hassett et al.:* low PDI (< 0.2) for stable vaccine-grade LNPs.	Simulated PDI < 0.2	Consistent with monodisperse, stable formulations suitable for large-scale production.	Kong et al., 2024; Hassett et al., 2021
Zeta potential (mV)	*Kong et al.:* −1.6 ± 0.4 (LNP-S) → near-neutral. Hassett et al.: mildly negative charge favored for immunotolerance and stability.	−1.0 to −4.4 mV (near-neutral)	Concordant near-neutral charge minimizing complement activation while maintaining uptake efficiency.	Kong et al., 2024; Hassett et al., 2021
PEG-lipid	*Zhang et al.:*	0.26 – 0.34	Simulated	Zhang et al., *Pharmaceutics*,

content (mol %)	optimal range 0.2–0.4 mol %, maximizing colloidal stability and mRNA delivery efficiency; PEG > 0.5 mol % reduces uptake, PEG < 0.1 mol % leads to aggregation.	mol % (mean ≈ 0.3 mol %)	PEG ratio matches experimental optima, confirming biological realism of the AI-optimized design window.	2023 (doi:10.3390/pharmaceutics17080950)
Encapsulation efficiency (%)	*Kong et al.:* 90–95 %; *Hassett et al.:* > 90 % for mRNA-LNP stability.	Not explicitly modeled, predicted high for ~90 nm, low-PEG formulations.	Qualitative agreement—simulated particles correspond to high-efficiency, stable designs.	Hassett et al., 2021; Kong et al., 2024

The in silico–optimized lipid nanoparticles closely reproduce experimentally validated physicochemical profiles across multiple studies, including the optimal ~90 nm particle size, near-neutral surface charge, and PEG-lipid content of ~0.3 mol %. Consistency with data from Hassett et al. (*Nat Nanotechnol*, 2021), Kong et al. (*J. Nanobiotechnol*, 2024), and Zhang et al. (*Pharmaceutics*, 2023) supports the translational robustness of the AI-guided LNP optimization pipeline.
