# Supplementary material for: A computational framework for optimizing mRNA vaccine delivery via AI-guided nanoparticle design and in silico gene expression profiling
Source: Front Immunol. 2025 Dec 5;16:1628583. doi: 10.3389/fimmu.2025.1628583 (PMC12714931; doi:10.3389/fimmu.2025.1628583)
Supplement: Supplementary file 5 [file Table2.pdf]

Component	Implementation	Details
Random Forest	randomForest (R, v4.7.1.2)	ntree = 500, mtry = 2, **5-fold cross-validation**, held-out performance **$R^2 \approx 0.91$ (validation set), low RMSE; 5-fold CV $R^2 \approx 0.87$ (training stability).**
Genetic Algorithm	GA::ga (R, v3.2.4)	popSize = 50, maxiter = 100, run = 50; fitness = RF-predicted ΔAUC
Sensitivity Analysis	Sobol' variance decomposition	Relative contributions to ΔAUC variance: **Size 0.41**, **Charge 0.32**, **PEG 0.17**, **Targeting 0.10**
Reproducibility	Seeds, exports, repository	set.seed(123); CSV intermedi e plot esportati; **tutto il codice e gli script R/Python su GitHub (link nel Data & Code Availability)**

Table S2. Machine-learning and optimization settings, sensitivity analysis, and reproducibility details.
